# Violence against women and unintended pregnancies in Nicaragua: a population-based multilevel study

**DOI:** 10.1186/1472-6874-14-26

**Published:** 2014-02-12

**Authors:** Mariano Salazar, Miguel San Sebastian

**Affiliations:** 1 Department of Public Health and Clinical Medicine, Epidemiology and Global Health, Umeå University, SE 901 85 Umeå, Sweden

**Keywords:** Epidemiology, Intimate partner violence, Nicaragua, Multilevel, Unintended pregnancies

## Abstract

**Background:**

Despite an increased use of contraceptive methods by women, unintended pregnancies represent one of the most evident violations of women’s sexual and reproductive rights around the world. This study aims to measure the association between individual and community exposure to different forms of violence against women (physical/sexual violence by the partner, sexual abuse by any person, or controlling behavior by the partner) and unintended pregnancies.

**Methods:**

Data from the 2006/2007 Nicaraguan Demographic and Health Survey were used. For the current study, 5347 women who reported a live birth in the five years prior to the survey and who were married or cohabitating at the time of the data collection were selected. Women’s exposure to controlling behaviors by their partners was measured using six questions from the WHO Multi-Country Study on Women’s Health and Domestic Violence against Women.

Area-level variables were constructed by aggregating the individual level exposures to violence into an exposure measurement of the municipality as a whole (n = 142); which is the basic political division in Nicaragua. Multilevel logistic regression was used to analyze the data.

**Results:**

In total, 37.1% of the pregnancies were reported as unintended. After adjusting for all variables included in the model, individual exposure to controlling behavior by a partner (AOR = 1.28, 95% CrI = 1.13–1.44), ever exposure to sexual abuse (AOR = 1.31, 95% CrI = 1.03–1.62), and ever exposure to physical/sexual intimate partner violence (AOR = 1.44, 95% CrI = 1.24–1.66) were significantly associated with unintended pregnancies. Women who lived in municipalities in the highest tertile of controlling behavior by a partner had 1.25 times higher odds of reporting an unintended pregnancy than women living in municipalities in the lowest tertile (AOR = 1.25, 95% CrI = 1.03–1.48).

**Conclusions:**

Nicaraguan women often experience unintended pregnancies, and the occurrence of unintended pregnancies is significantly associated with exposure to different forms of violence against women at both the individual and the municipality level. National policies aiming to facilitate women’s ability to exercise their reproductive rights must include actions aimed at reducing women’s exposures to violence against women.

## Background

Despite a global decline in total fertility rates [1] and increased use of contraceptive methods by women [2], unintended pregnancies represent one of the most evident violations of women’s sexual and reproductive rights around the world. It has been estimated that in 2008 four out of ten pregnancies that occurred worldwide were unintended. The lowest rate was in Oceania (34%) and the highest rate was in South America (64%) [3]. In a study describing the unintended pregnancy epidemic in the US, Finer and Kost [4] found that the unintended pregnancy rate varied from state to state and ranged from 40 to 65 unintended pregnancies per 1000 women aged 15–44 years. In Latin America and the Caribbean, 9,860,000 pregnancies were classified as unintended and represented 58% of the total pregnancies reported for the region in 2008 [3].

The epidemic of unintended pregnancies has been shown to affect the health of both women and their children [5], and women’s lack of control over their own reproductive cycle leads to increased morbidity and mortality [6-8]. Gipson et al. [9] reviewed evidence from 21 studies around the world and found a consistent association between unintended pregnancies and impaired mental health, poor antenatal care, diminished breastfeeding practices, increased infant mortality, and abortion-related morbidity and mortality [9].

### Factors associated with unintended pregnancies

Unintended pregnancies have been defined as “pregnancies that are reported to have been either unwanted (i.e., they occurred when no children, or no more children, were desired) or mistimed (i.e., they occurred earlier than desired)” [10]. In this study, pregnancy intentions were assessed only for those pregnancies ending in live births.

Unintended pregnancies can arise from the interplay between individual and social factors that limit contraceptive use or increase the rate of contraceptive failure. This is especially true for women living in low- and middle-income countries. A review by Campbell et al. [11] highlighted several barriers that hinder poor women’s ability to control their own fertility. These included a lack of access to healthcare facilities that provide family-planning services (including abortion), the high cost of contraceptives, and cultural factors that limit women’s agency and autonomy [11]. However, it must be noted that access to healthcare services does not necessarily ensure access to contraceptive technology. Health services might have a limited offering of contraceptive methods or the method offered (if any) might be influenced by the provider’s own prejudices [11].

A review by Black et al. [12] showed that almost half of all unintended pregnancies can be attributed to contraceptive failure, which is the combination of the inherent failure rate of a given method and the likelihood of the method being used correctly. They also showed that contraceptive failure is associated with both individual factors – such as young age, lack of experience using the method, alcohol and drug abuse, and lack of information about sexual and reproductive health – and relationship factors such as intimate partner violence (IPV).

### Intimate partner violence and men’s control over women’s fertility

Violence against women (VAW) is often used in patriarchal societies to protect men’s power status in society and to maintain women’s subordination [13,14]. In many societies, women are most likely to experience violence from someone close to them such as their intimate partners. Thus, IPV is one of the most common forms of violence experienced by women. For example, the WHO Multi-Country Study on Women’s Health and Domestic Violence Against Women has estimated that between 15% and 75% of all women have experienced physical or sexual abuse by a current or former partner [15]. Recent data from the UN Multi-Country Cross-Sectional Study on Men and Violence in Asia and the Pacific found that between 4% and 41% of the men interviewed reported having ever engaged in physical or sexual violence against a current or former partner [16].

Physical IPV is often associated with men’s attempts to limit women’s agency and autonomy [15,16], and this controlling behavior also affects women’s reproductive lives. Men who are violent toward their female partners can interfere with their partner’s reproductive autonomy by forcing them to have sex, by destroying or disabling their contraceptive methods, by interfering with their access to health care [17], or by deciding which contraceptive method to use (or not to use) [10]. Thus, it is not surprising that a number of studies from around the world have identified a strong association between IPV and unintended pregnancies [18-20]. For example, data from the WHO Multi-Country Study on Women's Health and Domestic Violence Against Women showed that women exposed to IPV had 1.69 times higher odds of reporting an unintended pregnancy than those not exposed to IPV even after adjusting for confounding factors [21]. The authors of that study suggested that reducing IPV exposure by 50% could decrease unintended pregnancies by up to 18% in some settings [21].

### Rationale

Women’s experiences with unintended pregnancies must be understood beyond individual practices and beliefs. Thus, researchers must take into account the contextual factors – such as access to contraceptive services and the gender power structures in society – that shape women’s ability to exercise their reproductive rights [10,22]. Although quantitative studies have assessed the relationship between IPV and unintended pregnancies [4,18-21], few have attempted to include in their analysis the measurement of contextual factors that influence women’s fertility [23-25] and even fewer have included community-level measures of VAW [22]. In a pioneering population-based study assessing the relationship between community-level measures of IPV and unintended pregnancies among Colombian women, Pallito and O’Campo found that the odds of having an unintended pregnancy were higher in settings with high levels of patriarchal control and high rates of IPV [22].

In Nicaragua, women have experienced a decrease in their total fertility rate from 4.2 children per woman in 1998 to 2.8 in 2006 [26]. In addition, seven out of ten partnered women use a modern method of contraception [26]. In spite of these achievements, Nicaraguan women continue to face reproductive challenges. The teenage pregnancy rate is high (106 births per 1000 women aged 15–19 years), 14% of the total fertility rate is due to unintended pregnancies, and 10% of the women have an unmet need for family planning services [26]. In addition, half of all Nicaraguan women have experienced physical IPV at some point [27] and 40% of those exposed to IPV have experienced a continuous pattern of abuse [28]. Until now, no studies have assessed the relationship between exposure to different forms of VAW and unintended pregnancies among Nicaraguan women. This study aims to measure the association between individual and community exposure to different forms of VAW (physical/sexual IPV by the partner, sexual abuse by any person, or controlling behavior by the partner) and unintended pregnancies. Specifically, this study aims to determine if the rate of unintended pregnancies varies in communities with low levels of VAW compared with those with medium and high levels of VAW.

## Methods

### Study design

Data from the 2006/2007 Nicaraguan Demographic and Health Survey (NDHS) were used. The survey collects cross-sectional data on household demographic characteristics as well as a wide range of data covering children’s health outcomes and women’s sexual and reproductive health [26].

The NDHS uses a multi-stage cluster sampling strategy to interview women from 142 urban and rural municipalities across the country. Detailed information on the sampling procedures conducted for this survey can be found elsewhere [26]. For the current study, 5347 women who reported a live birth in the five years prior to the survey and who were married or cohabitating at the time of the data collection were selected.

### Measurements

#### Individual variables

Exposure to physical/sexual IPV was measured using a modified version of the Conflict Tactics Scale, an instrument designed to ask women about exposure to different violent acts by their current or former partners [29]. A woman was considered to have experienced physical/sexual IPV if she reported ever being pushed, slapped, punched, kicked, dragged by the hair, burned, threatened with a weapon, or physically forced to have intercourse by a current or former partner.

Women’s exposure to controlling behaviors by their partner was measured using six questions from the WHO Multi-Country Study on Women’s Health and Domestic Violence Against Women [30]. These questions measured men’s controlling behavior such as limiting a woman’s contact with family, friends, or health care; insisting on knowing where a woman is at all times; getting upset if a woman talks to other men; or always suspecting a woman of infidelity. A woman was considered to have been exposed to controlling behavior if she answered yes to any of the six questions described above.

Exposure to sexual violence by any person was measured using the following two questions: “Have you ever been forced to have penetrative sex” and “Have you ever been forced to perform other sexual acts (touching someone, undressing, touching yourself, etc.) against your will?” Answering yes to either of these questions was considered exposure to sexual violence.

Ever exposure to physical/sexual IPV (Cronbach’s alpha value of 0.88) and controlling behavior by a partner (Cronbach’s alpha value of 0.80) showed good reliability. Moderate inter-item reliability was found for the two items that measure exposure to sexual violence (Cronbach’s alpha value of 0.51).

Principal component analysis was used to create a household socioeconomic status index that served as a proxy for women’s socioeconomic situation. This is a technique that has been widely used to construct socioeconomic indexes using asset information [31]. The index was constructed from 18 questions assessing if the household had several assets, including different electronic devices (radio, iron, computer, etc.), and if the household had any form of transportation such as cars and bicycles among others. Once the index was created, it was categorized into quintiles.

Ever use of modern contraception was defined as a woman using any form of contraceptive method apart from periodic abstinence, withdrawal, calendar-based methods, or lactational amenorrhea methods. Other individual variables collected in the survey were the women’s age (years) and education level (no education, primary school, high school, or college and higher).

#### Municipality-level variables

Nicaragua is divided into 15 departments and 2 autonomous regions, and the departments and autonomous regions are further divided into 142 municipalities. Seven municipality-level variables were constructed, six of which were created by aggregating individual-level exposures. Five variables represent the percentage of women in each municipality that 1) were ever exposed to sexual violence by any person, 2) were ever exposed to physical/sexual IPV, 3) were ever exposed to controlling behavior by a partner, 4) ever used modern contraceptives, and 5) were illiterate. The sixth variable measured the percentage of the population in each municipality living in extreme poverty (defined as the percentage of the population in the lowest socioeconomic quintile). These variables were categorized into tertiles. The seventh variable identified the place of residency (urban vs. rural).

### Analysis

Univariate, bivariate, and multivariate statistics were used to describe the data. Sampling weights were introduced in the bivariate analyses to compensate for the women’s unequal selection probability due to the sampling method used to collect the data [26]. Sampling weights were calculated by multiplying the household’s selection probability by the woman’s selection probability. Women’s selection probability was inversely proportional to the number of women of childbearing age in the home, and detailed information on this can be found elsewhere [26]. The results of the bivariate analyses are presented as weighted means or percentages. Student’s *t*-test and chi-squared test were used to assess differences between groups.

A multilevel logistic analysis was conducted using runmlwin [32], a program designed to run multilevel models from within the Stata12 software package (StataCorp, College Station, Texas). All variables were included in the multivariate and multilevel analyses.

Two analytical strategies were used. First, a Bayesian estimation using a Markov chain Monte Carlo (MCMC) method was used to obtain adjusted odds ratios (AORs) and 95% credible intervals (CrI). MCMC was used because quasi-likelihood methods used to model multilevel analyses have been reported to be biased [32,33]. However, a disadvantage of MCMC is that it does not allow sampling weights to be included in the analysis. In order to compare if different results arose from including sampling weights in the analysis, the results from a second-order penalized quasi-likelihood linearization (PQL2) are also presented. Sampling weights included in the final analysis were scaled by creating new weights that added to the cluster sample size. This technique has been described elsewhere [34].

Both analytical techniques are presented in three consecutive models. The first model was an empty model that only included a random intercept and the outcome variable of unintended pregnancies. This model aimed to assess if the occurrence of unintended pregnancies was influenced by differences between municipalities. The second model added individual-level variables to determine if any municipality differences were due to variations in women’s characteristics within each municipality. The third model included both individual-level and municipality-level variables.

Multicolinearity between the variables included in the models was assessed using tolerance variance inflation factor (VIF) post-estimation diagnostic tests. Multicolinearity was defined as tolerance values below 0.1 and VIF values equal to or greater than 10. No multicolinearity between the variables was found (tolerance = 0.92 and VIF = 1.01).

Two measures of variation between municipalities were used. The variance partition coefficient (VPC) represents the proportion of the variance (VA) that is due to the difference between municipalities and is calculated as follows: VPC = VA/(VA + 3.29) [33]. The median odds ratio (MOR) is defined as “the median value of the odds ratio between the area at highest risk and the area at lowest risk when randomly picking out two areas” [33]. The MOR was computed using the following formula: $\mathrm{MOR}\;=\;\exp\;\left(0.95\sqrt{\mathrm{VA}}\right)$[33].

### Ethical considerations

This manuscript used a dataset that was freely available from online sources (http://ghdx.healthmetricsandevaluation.org/record/nicaragua-reproductive-health-survey-2006-2007). The dataset does not contain any individual identifiers that would make it possible to track women’s identities. The study is in compliance with the Helsinki Declaration and it was approved by the Nicaraguan National Autonomous University of León Ethics Committee.

## Results

### The prevalence of unintended pregnancies and women’s characteristics

As described earlier, unintended pregnancies include both unwanted and mistimed pregnancies. In total, 24% of the pregnancies were reported as mistimed, 13.5% as unwanted, and 37.1% as unintended. In the bivariate analysis, few significant differences were found between women who reported unintended pregnancies in the last five years and those who did not. Women who reported unintended pregnancies were mainly from urban environments, had a low socioeconomic status, and reported having used modern contraceptive methods (Table 1, p < 0.05). Women who reported unintended pregnancies had significantly higher exposures to physical/sexual IPV, sexual abuse, and controlling behavior by a partner than those not reporting unintended pregnancies (Table 1, p < 0.05).

**Table 1:** Demographic characteristics of the women in this study (n = 5347) and their exposures to different forms of violence against women stratified by the occurrence of unintended pregnancy

**Characteristic**	**Unintended pregnancies no n = 3413**	**Unintended pregnancies yes n = 1934**	**All n = 5347**
	**%**	**%**	**%**
Residency (Rural)*	55.3	46.7	52.1
Age in years, Mean (SE)	27.0 (0.15)	27.6 (0.20)	27.2 (0.12)
Education			
No education	16.6	16.6	16.6
Primary School	43.0	42.5	42.8
High school	28.7	31.3	29.7
College or higher	11.5	9.4	10.7
Socioeconomic status*			
Quintile 1	30.4	25.2	28.5
Quintile 2	21.4	22.5	21.8
Quintile 3	16.7	22.8	18.9
Quintile 4	15.5	17.5	16.2
Quintile 5	15.8	11.8	14.3
Ever use of modern contraceptives			
(yes)*	93.0	95.4	93.9
Controlling behavior by			
partner (yes)*	47.5	57.6	51.3
Ever sexual abuse by any person			
(yes)*	7.3	12.6	9.3
Ever physical/sexual IPV			
(yes)*	19.9	30.4	23.8

*t-test or Chi-squared test, p < 0.05. IPV = Intimate partner violence.

Weighted percentages are shown.

### Municipality-level characteristics

Each municipality had an average of 37.7 women sampled with a minimum of 4 and a maximum of 370 women. The means and standard errors (SE) of the municipality-level variables are shown in Table 2.

**Table 2:** Descriptions of the municipality-level variables

**Characteristic**	**Municipalities n = 142**	
	**Mean (SE)**	**Range**
% of women exposed to sexual violence by any person	9.4 (0.35)	0–23.7
% of women exposed to physical/sexual IPV	27.5 (0.65)	0–54.2
% of women using modern contraceptives	68.6 (0.68)	33.3–100
% of the population in extreme poverty	42.8 (1.57)	13.5–87.4
% of women who are illiterate	25.6 (1.12)	7.7–65
% of women exposed to controlling behavior by a partner	51.1 (0.79)	26.7–86.4

IPV = Intimate partner violence.

### Multilevel analysis

The results of the multilevel analysis are shown for the MCMC method in Table 3 and for the PQL2 model with scaled sampling weights in Table 4.

**Table 3:** Unweighted multilevel logistic regression of the association between unintended pregnancies and individual-level and municipality-level characteristics among Nicaraguan women

	**1. Empty model**	**2. Model with individual-level variables**		**3. Model with individual-level and municipality-level variables**	
Measures of association (AOR, 95% CrI)*					
*Individual-level variables*					
Controlling behavior by a partner (yes)	---	1.29	(1.13–1.47)	1.28	(1.13–1.44)
Ever sexual abuse by any person (yes)	---	1.32	(1.06–1.62)	1.31	(1.03–1.62)
Ever physical/sexual IPV (yes)	---	1.45	(1.24–1.68)	1.44	(1.24–1.66)
*Municipality-level variables*					
% of women exposed to controlling behavior by a partner					
Lowest tertile	---	---	---	1.00	
Middle tertile	---	---	---	1.10	(0.89–1.33)
Highest tertile	---	---	---	1.25	(1.03–1.48)
% of women exposed to sexual violence by any person					
Lowest tertile	---	---	---	1.00	
Middle tertile	---	---	---	1.05	(0.84–1.28)
Highest tertile	---	---	---	1.00	(0.75–1.33)
% of women exposed to physical/sexual IPV					
Lowest tertile	---	---	---	1.00	
Middle tertile	---	---	---	1.09	(0.90–1.32)
Highest tertile	---	---	---	1.20	(0.92–1.55)
**Measures of clustering**					
Area-level variance (SD)	0.081 (0.026)	0.066 (0.025)		0.062 (0.029)	
PCV	---	−18.5%		−6%	
MOR	1.31	1.27		1.26	
VPC	0.024	0.019		0.018	

*Adjusted by residency, age, education, socioeconomic status, ever use of modern contraceptive methods, % of women using modern contraceptive methods at the municipality level, % of women illiterate at the municipality level, and % of the population living in extreme poverty at the municipality level. PCV = percentage of change in variance. MOR = median odds ratio. VPC = variance partition coefficient.

Bayesian estimates of the measures of association and clustering are shown. n = 5347.

**Table 4:** Weighted multilevel logistic regression of the association between unintended pregnancies and individual-level and municipality-level characteristics in Nicaraguan women

	**1. Empty model**	**2. Model with individual-level variables**		**3. Model with individual-level and municipality-level variables**	
Measures of association (AOR, 95% CrI)*					
*Individual-level variables*					
Controlling behavior by a partner (yes)	---	1.28	(1.10–1.50)	1.26	(1.08–1.47)
Ever sexual abuse by any person (yes)	---	1.26	(0.99–1.61)	1.25	(0.98–1.60)
Ever physical/sexual IPV (yes)	---	1.44	(1.21–1.71)	1.43	(1.20–1.70)
*Area-level variables*					
% of women exposed to controlling behavior by a partner					
Lowest tertile	---	---	---	1.00	
Middle tertile	---	---	---	1.08	(0.86–1.35)
Highest tertile	---	---	---	1.27	(1.06–1.53)
% of women exposed to sexual violence by any person					
Lowest tertile	---	---	---	1.00	
Middle tertile	---	---	---	1.04	(0.84–1.29)
Highest tertile	---	---	---	1.10	(0.88–1.37)
% of women exposed to physical/sexual IPV					
Lowest tertile	---	---	---	1.00	
Middle tertile	---	---	---	1.03	(0.84–1.25)
Highest tertile	---	---	---	1.10	(0.84–1.43)
**Measures of clustering**					
Area-level variance (SD)	0.095 (0.027)	0.079 (0.025)		0.054 (0.022)	
PCV	---	−16%		−31%	
MOR	1.34	1.30		1.24	
VPC	0.028	0.023		0.016	

*Adjusted by residency, age, education, socioeconomic status, ever use of modern contraceptive methods, % of women using modern contraceptive methods at the municipality level, % of women illiterate at the municipality level, and % of the population living in extreme poverty at the municipality level. PCV = percentage of change in variance. MOR = median odds ratio. VPC = variance partition coefficient.

Second-order penalized quasi-likelihood linearization estimates and measures of association and clustering are shown. n = 5347.

#### MCMC method

Model 1 (the empty model) showed that 2.4% of the unintended pregnancies could be explained by variation in the municipality’s characteristics (Table 3). It also showed that in the median case the residual heterogeneity between municipalities resulted in 1.31 times greater odds of a woman reporting an unintended pregnancy when randomly picking two women from different areas (Table 3).

When individual-level variables were included in the model (Model 2), the municipality-level variance decreased by 18.5%. This illustrated that some of the area-level variance could be explained by variations in women’s individual characteristics within each municipality (Table 3). Consequently, the MOR and the VPC also decreased. This model shows that exposure to controlling behavior by a partner, ever exposure to sexual abuse, ever exposure to physical/sexual IPV, women’s age, and ever use of contraceptive methods were associated with unintended pregnancies after adjusting for demographic factors (Table 3, p < 0.05).

In Model 3, the area-level variance decreased by 6% compared to the value found in Model 2. Both the VPC and the MOR decreased as well. The VPC showed that 1.8% of the unintended pregnancies could be attributed to differences at the municipality level (Table 3). This model also found that in the median case the residual heterogeneity between municipalities resulted in 1.26 times greater odds of a woman reporting an unintended pregnancy when randomly picking two women from different areas (Table 3). After adjusting for all variables included in the model, individual exposure to controlling behavior by a partner (AOR = 1.28, 95% CrI = 1.13–1.44), ever exposure to sexual abuse (AOR = 1.31, 95% CrI = 1.03–1.62), and ever exposure to physical/sexual IPV (AOR = 1.44, 95% CrI = 1.24–1.66) were significantly associated with unintended pregnancies (Table 3).

At the municipality level, the percentage of women exposed to controlling behavior by a partner was associated with the occurrence of unintended pregnancies. Women who lived in municipalities in the highest tertile of controlling behavior by a partner had 1.25 times higher odds of reporting an unintended pregnancy than women living in municipalities in the lowest tertile. This difference was seen even after adjusting for all individual and other area-level variables (Table 3). No significant association was found between unintended pregnancies and the percentage of women exposed to sexual violence by any person or the percentage of women ever exposed to physical/sexual IPV at the municipality level (Table 3, p > 0.05).

#### PQL2 method

There were minimal differences in area-level variances between the PQL2 and MCMC methods (Table 4). However, in the weighted PQL2 analysis the individual exposure to sexual abuse by any person was found to be non-significant (AOR = 1.25, 95% CrI = 0.98–1.60) (Table 4), which was the opposite of what was found in the MCMC analysis (AOR = 1.31, 95% CrI = 1.03–1.62) (Table 3).

## Discussion

This study shows that individual exposure to controlling behavior by a partner and to physical/sexual IPV significantly increased women’s adjusted odds of reporting unintended pregnancies during the five years prior to the data collection. Exposure to sexual abuse by any person was found to be significantly associated in the model using the MCMC method of analysis but not significantly associated in the model using the PQL2 method. At the municipality level, both models showed that women living in municipalities in the highest tertile of controlling behavior by a partner had higher odds of reporting an unintended pregnancy than women living in municipalities in the lowest tertile even after adjusting for individual and municipality level variables.

Individual exposure to the different forms of VAW identified in this paper can influence the occurrence of unintended pregnancies in different ways. Our findings on the relationship between unintended pregnancies and individual exposure to sexual abuse by any person are mixed and depend on the method of analysis. However, we argue that this relationship must still be discussed because both methods showed a positive association even though the relationship in the PQL2 method was not statistically significant. Sexual abuse can interfere with women’s reproductive autonomy in many ways. In an article analyzing the evidence from 14 review papers, Maniglio found a consistent association between sexual abuse during childhood and high risk sexual behaviors such as having multiple partners and having unprotected sex [35]. Sexual abuse can also significantly impair women’s mental health [35], and this in turn can affect their ability to negotiate the use of contraception or to correctly use a given contraceptive method.

Our finding that individual exposure to physical/sexual IPV increases the odds of reporting unintended pregnancies is consistent with other studies conducted in other countries [5,18-21]. We also found that both physical/sexual IPV and controlling behavior by a partner remained significantly associated with unintended pregnancies when they were included in the same multilevel model. This suggests that these intertwined but different forms of violence can hinder women’s reproductive autonomy through different pathways. Physical/sexual IPV has been shown to affect women’s mental health [36] and diminish their self-esteem [27], and this can impair their ability to negotiate contraception [10] as well as increase the likelihood of contraceptive failure. On the other hand, controlling behavior by a partner can interfere with women’s reproductive freedom through more direct pathways. First, it can hinder women’s contraceptive use by limiting their access to health care [17]. Second, it can facilitate contraceptive failure by forcing women to have sex or by destroying or disabling their contraceptives [17].

Researchers have highlighted that women’s fertility is often shaped by contextual and cultural factors beyond their control [10,22]. Our study reinforces these findings by showing that a high level of exposure to controlling behavior by a partner at the municipality level is associated with the occurrence of unintended pregnancies among partnered women. Women’s individual exposure to violence has been found to be strongly linked to contextual factors [37]. In our study it is possible, therefore, that the community levels of controlling behavior by a partner have an indirect influence on unintended pregnancies by increasing women’s individual exposure to different forms of VAW.

The random effect results highlight that municipality levels of controlling behavior by a partner contributed to less than 2% of the total unintended pregnancies among Nicaraguan women who have a partner. Although controlling behavior at the municipality level was associated with unintended pregnancies, the variation between municipalities was very low. Thus, interventions aimed at curtailing unintended pregnancies should target all women and not just those in specific municipalities.

### Limitations and strengths

The cross-sectional design of this study allowed us to determine associations but did not allow us to determine a causal link between exposures and outcomes. Also, measuring unintended pregnancies (including those that were mistimed and unwanted) using demographic survey data is challenging, and recall bias can arise when asking women retrospectively about former pregnancies [10]. In addition, demographic surveys traditionally only include questions about pregnancy intentions for those pregnancies ending in live births, not for those ending in abortions [10]. This is also true for the data used in this paper. Thus, it is possible that the prevalence of unintended pregnancies in this study is an underestimation of the true figure in this population.

As with unintended pregnancies, inquiring about VAW in demographic surveys is also a challenge. Asking about VAW requires certain conditions such as privacy, time, and confidentiality that might be difficult to achieve when questions about VAW are asked as a small part of a more extensive survey [38]. Thus, it is possible that the different VAW measures used in this paper are an underestimation of the true prevalence.

This study also has significant strengths. The external validity of this study is strengthened by its nationwide population-based design. Also, the multilevel analyses were conducted using both MCMC and PQL2 methods and this allowed us to compare both weighted and unweighted results. In addition, the models presented here include multiple forms of VAW both at the individual and at the community level.

## Conclusions

Nicaraguan women often experience unintended pregnancies, and the occurrence of unintended pregnancies is significantly associated with exposure to different forms of VAW at both the individual and the municipality level. It is clear, therefore, that national policies aiming to facilitate women’s ability to exercise their reproductive rights must include actions aimed at reducing women’s individual exposures to VAW. These policies must also include strategies that challenge the exercising of control over women as a sign of masculinity in Nicaragua.

## Competing interests

The authors declare that they have no competing interests.

## Authors’ contributions

MS conceived the study, participated in its design, performed the statistical analysis and drafted the manuscript. MSS performed the statistical analysis and drafted the manuscript. Both authors read and approved the final manuscript.

## Pre-publication history

The pre-publication history for this paper can be accessed here:

http://www.biomedcentral.com/1472-6874/14/26/prepub
